# Incident Cardiometabolic Comorbidities in Smokers with/Without Chronic Obstructive Pulmonary Disease: A Long-Term Cohort Study

**DOI:** 10.3390/jcm13247627

**Published:** 2024-12-14

**Authors:** Beatriz Herrero-Cortina, Aura Maldonado-Guaje, Jorge Rodriguez-Sanz, Ana Boldova-Loscertales, Pablo Cubero-Marin, Marta Marin-Oto, David Sanz-Rubio, Jose M. Marin

**Affiliations:** 1 Precision Medicine in Respiratory Diseases Group, Hospital Universitario Miguel Servet, Instituto de Investigación Sanitaria (IIS) de Aragón, 50009 Zaragoza, Spain; beafisiorespi@gmail.com (B.H.-C.); aura.maldonado@gmail.com (A.M.-G.); jrsanz265@gmail.com (J.R.-S.); jpcuberomarin@gmail.com (P.C.-M.); marta.marin.oto@gmail.com (M.M.-O.); davidsanzrubio91@gmail.com (D.S.-R.); 2 Nursing Department, Universidad San Jorge, 50830 Zaragoza, Spain; 3 Pulmonary Division, Hospital Universitario Miguel Servet, 50009 Zaragoza, Spain; 4 Pulmonary Division, Hospital Royo Villanova, 50015 Zaragoza, Spain; anabl81@yahoo.es; 5 Pulmonary Division, Hospital Clínico Universitario Lozano Blesa, 50009 Zaragoza, Spain; 6 Department of Medicine, University of Zaragoza, 50009 Zaragoza, Spain; 7 CIBER de Enfermedades Respiratorias, Instituto Salud Carlos III, Ministry of Health, 28071 Madrid, Spain

**Keywords:** COPD, smokers, lung function, cardiometabolic multimorbidity

## Abstract

**Backgrounds:** Despite the significant global health impact of cardiometabolic multimorbidity (CMM), our understanding of potential predictors associated with its development in smokers, remains limited. Objective: This study aimed to investigate whether a new COPD diagnosis and the rate of lung function decline serve as predictors for incident CMM (defined as having at least two of the following comorbidities: cerebro-cardiovascular diseases, hypertension, dyslipidemia, and diabetes mellitus) in smokers. **Methods:** An observational longitudinal analysis of prospectively collected data was conducted, including smokers without a previous COPD diagnosis and any cardiometabolic conditions. Sociodemographic and clinical data (body mass index, smoking history, respiratory symptoms, and hospital admissions) were collected at baseline. Lung function tests were performed at baseline and at the end of the follow-up period. The incidence of CMM, a new positive diagnosis of COPD, and the forced expiratory volume in 1 s (FEV_1_) annual rate of decline were prospectively registered. Adjusted Cox proportional hazard models were adopted to explore risk factors associated with the incidence of CMM. **Results:** From the 391 smokers included in the study, 207 (53%) were newly diagnosed with COPD, and 184 had a preserved spirometry at baseline (non-COPD group). After nearly a decade of follow-up, 34% (n = 133) of smokers developed CMM. This group was characterized by male predominance, older age, higher BMI and pack-years of smoking, lower post-FEV_1_, baseline COPD diagnosis, and a history of hospital admission. A positive diagnosis of COPD at baseline and a greater rate of lung function decline (ΔFEV_1_ ≥ 40 mL/year) were independent predictors for developing CMM. **Conclusions:** A new COPD diagnosis and an accelerated decline in lung function are significantly associated with the development of CMM in smokers.

## 1. Introduction

Currently, chronic obstructive pulmonary disease (COPD) is the most prevalent chronic respiratory disease in adults [1], remains a leading cause of disability and death, and is associated with a substantial economic burden [2]. COPD-attributable costs increase with disease severity, with hospitalizations, symptom burden, and comorbidities serving as the primary drivers of these expenses [3,4]. For these reasons, investments in the identification of individuals at risk of developing COPD are necessary to implement early interventions and reduce the economic cost in the future by slowing disease progression [5].

The main risk factor for the development of COPD is the inhalation of toxic particles, especially tobacco smoke [6]. These factors are also associated with the development of cancer and metabolic and cardiovascular diseases [7,8]. Therefore, it is not surprising that many smokers develop several of these diseases simultaneously. We previously demonstrated in a large population-based cohort that those with COPD had more comorbidities at a younger age compared to controls [9]. A comparison of COPD and non-COPD cohorts across five incremental age groups showed that the number of comorbidities, including cardiometabolic comorbidities, in COPD patients was similar to that of non-COPD patients 15 to 20 years older. The findings persisted after adjusting for smoking [9]. However, this topic remains controversial since a cross-sectional case–control study reported that the prevalence of cardiometabolic comorbidities was similar between newly diagnosed COPD patients and non-COPD smokers [10]. It has been proposed that COPD may be a coexisting disease of multimorbidity disorders [11]. This hypothesis is of great importance because it would identify a common pathobiological process with effects at the level of different tissues and organs and the need for a specific treatment.

Multimorbidity increases the risk of hospitalization and polypharmacy and negatively impacts survival in COPD [12,13]. Specifically, cardiovascular and metabolic comorbidities were associated with higher excess costs in this population [4,14]. The coexistence of at least two cardiometabolic comorbidities (e.g., cerebro-cardiovascular diseases, hypertension, diabetes, and dyslipidemia) has been described by some authors as “cardiometabolic multimorbidity” (CMM), although there is no consensus on this definition [15]. Longitudinal studies exploring the factors associated with the development of CMM in smokers with or without COPD remain understudied. More importantly, whether the progression of the pathological and functional impairment of the respiratory system occurs in parallel with incident cardiometabolic comorbidities is unknown. This information is essential to finally identify potential biomarkers of progression of CMM in smokers.

In this long-term longitudinal study of a cohort of smokers without baseline comorbidities, we evaluated whether the concomitant diagnosis of COPD is a risk predictor for incident CMM and whether lung function decreases in parallel with the appearance of these new comorbid conditions.

## 2. Methods

### 2.1. Study Design and Participants

**Figure 1:**
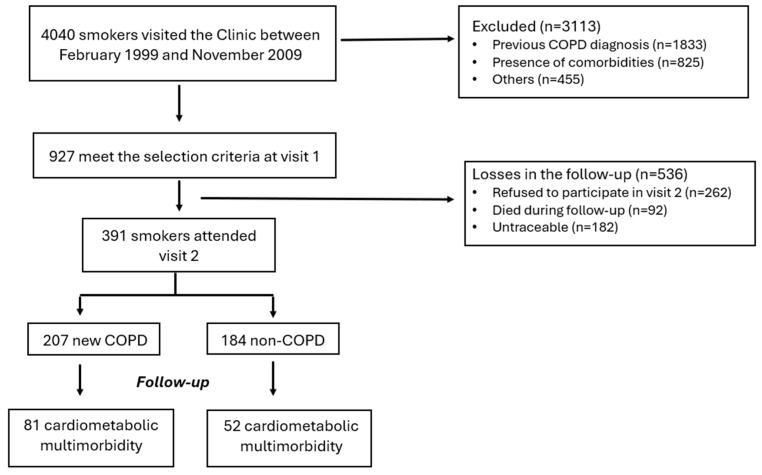
Flowchart of participants throughout the study.

This is an observational longitudinal analysis of prospectively collected data from smokers recruited at the Smoker Clinic in the Hospital Miguel Servet in Zaragoza (Spain) as part of the BODE International Cohort Study designed to understand the natural history of COPD and smokers without COPD [16]. Participants who had current or former tobacco use for longer than 20 pack-years were enrolled between February 1999 and November 2009. For the purpose of this analysis, participants were excluded if they had undergone previous thoracic surgery or were diagnosed with alpha-1 antitrypsin deficiency, as these could influence baseline spirometric values and accelerate lung function decline. Patients with active or suspected cancer prior to recruitment or during the follow-up period were also excluded due to potential impacts on outcomes. Additionally, those with any concomitant chronic morbid condition other than COPD under treatment, including previous cardiovascular (CVS) events (e.g., myocardial infarction, stroke, or other CVS hospitalization) and known major CVS risk factors (e.g., hypertension, dyslipidemia, and diabetes mellitus) were also excluded to ensure the sample did not present any cardiometabolic comorbidities or associated risk factors (Figure 1). In-person second visit was performed at the clinic after at least 5 years of recruitment of each subject (visit 2). The Aragon Institute of Health Research approved the study (IRB.03/15), and all participants gave written informed consent.

### 2.2. Clinical Measures

At baseline (visit 1), sociodemographic (gender and age) and clinical data (body mass index (BMI) in kg/m^2^), smoking history (smoking status and pack-years), and respiratory symptoms such as chronic cough and dyspnea were initially registered. Dyspnea was stratified according to the modified Medical Research Council dyspnea scale [17]. Chronic bronchitis was defined as cough and phlegm for at least 3 months per year for at least 2 consecutive years [18]. Spirometry and diffusing capacity of the lungs for carbon monoxide tests were performed in accordance with the American Thoracic Society/European Respiratory Society (ATS/ERS) guidelines [19,20]. Post-bronchodilator forced expiratory volume in 1 s (FEV_1_) was expressed as percentages of predicted values [21]. COPD diagnosis was defined as individuals over 40 years of age who exhibited a post-bronchodilator FEV_1_/forced vital capacity ratio of less than 0.7. Additionally, the post-bronchodilator FEV_1_ was also used to classify these patients according to the Global Strategy for the Diagnosis, Management, and Prevention of COPD, GOLD classification [6]. Participants diagnosed with new cases of COPD were referred to a specialized clinic to receive appropriate therapy tailored to their condition. The occurrence of hospital admissions for respiratory problems prior to recruitment was also recorded.

### 2.3. Follow-Up and Lung Function Decline

Regardless of the clinical management performed on each participant, all subjects were scheduled for another in-person evaluation (visit 2) at least 5 years after visit 1. Between visit 1 and visit 2, a new positive diagnosis of COPD and the development of cardiometabolic comorbidities (both based on a physician’s diagnosis) were prospectively recorded by reviewing the electronic medical records every year. All-cause mortality was recorded, using information obtained from the family, and then confirmed by reviewing medical records as published previously [16]. At visit 2, all clinical procedures performed at visit 1 were repeated. At this point, all new health events and new cardiometabolic morbidities were ascertained. For the purpose of this analysis, CMM was defined as having at least two of the following comorbidities: cerebro-cardiovascular diseases (including primarily stroke, arrhythmia, heart failure, peripheral vascular disease, and coronary artery disease), hypertension, dyslipidemia, and diabetes mellitus.

The post-bronchodilator FEV_1_ decline was calculated by subtracting FEV_1_ at visit 2 from FEV_1_ at baseline and by dividing by follow-up time in years to obtain the annual rate of decline expressed in mL/year (ΔFEV_1_). Participants were dichotomized as greater decliners (ΔFEV_1_ ≥ 40 mL/year) or lesser decliners (ΔFEV_1_ < 40 mL/year), according to ECLIPSE (Evaluation of COPD Longitudinally to Identify Predictive Surrogate End-Points) data [22].

### 2.4. Statistical Analysis

Descriptive statistics were used to summarize demographic and clinical features. Baseline was taken as the measurement at the initial visit. Differences between patients based on a positive COPD diagnosis at baseline (COPD group vs. non-COPD group) and the development of CMM (≥2 cardiometabolic comorbidities vs. ≤1 cardiometabolic comorbidity) during the follow-up were analyzed using Student’s *t*-test for independent samples, as well as Mann–Whitney and chi-squared test or Fisher´s exact test as appropriate. The risk of incident CMM was analyzed using the Kaplan–Meier survival function per COPD diagnosis at baseline (COPD group vs. non-COPD group), and the log-rank test was applied to compare the groups. The association between the baseline diagnosis of COPD, demographics variables, and FEV_1_ rate of decline with each incident morbidity of interest (e.g., cerebro-cardiovascular diseases, hypertension, dyslipidemia, and diabetes mellitus) or CMM was assessed with Cox proportional hazard regression analyses adjusted by age, sex, and body mass index (BMI). The results are expressed as hazard ratios (HRs) with 95% confidence intervals (CIs). The proportionality assumption was checked with the Schoenfeld test. A threshold of *p* < 0.05 was considered significant. Statistical analyses were conducted using Stata version 17 (StataCorp, College Station, TX, USA) and Jamovi version 2.3.21.0 (The Jamovi Project, 2023).

## 3. Results

Among the smokers attending our outpatient clinics between February 1999 and November 2009, 927 met the selection criteria (visit 1). After a median (interquartile range (IQR)) follow-up of 9.5 (6.75–13.0) years, 391 attended in-person visit 2 at the clinic, while 262 declined to participate in this visit, 182 were untraceable, and 92 died (Figure 1). At baseline, participants who did not complete visit 2 were older; had more dyspnea, as quantified by the mMRC score; and had worse lung function (Table S1, Supplementary Materials). Among those smokers who attended visit 2, 207 were newly diagnosed with COPD, and 184 had a preserved spirometry (non-COPD) at baseline. Patients with COPD were older compared to non-COPD participants (mean (SD), 61 (8) vs. 51 (11) years, *p* < 0.001), mostly men (91% vs. 71%, <0.001), had a significant history of smoking (47.4 (22.4) vs. 33.9 (18.9) pack-year, *p* < 0.001), and more participants required at least one hospital admission the previous year due to respiratory symptoms (40% vs. 27%, *p* = 0.032) (Table 1). Most patients with COPD had mild-to-moderate disease severity (n = 168, 81%), as defined by the GOLD recommendations [6].

### 3.1. Rate of Lung Function Annual Decline and New COPD Diagnosis During Follow-Up

From baseline to visit 2, the mean decline in FEV_1_ was 33 mL/y among smokers without COPD and 31 mL/y in those with COPD (mean difference: 2.4 mL/year; 95% CI: −10.5 to 5.72 mL/y, *p* = 0.556). Overall, almost 41% of participants were considered greater FEV_1_ decliners (ΔFEV_1_ ≥ 40 mL/year) with similar proportions of greater decliners in both groups (Table S2, Supplementary Materials). Greater decliners had higher FEV_1_ and DL_CO_ values at baseline, but otherwise, the two groups did not show any differences in demographics, anthropometrics, smoking history, prevalence of respiratory symptoms, or previous hospital admissions due to respiratory symptoms. In the non-COPD group, 73 (40%) individuals at baseline met the criteria for pre-COPD and after the follow-up, 29 (16%) of them developed COPD (Table S3, Supplementary Materials). After adjusting for age, sex, BMI, pack-years of smoking, and current smoking status, having one or more hospital admissions due to respiratory symptoms during the previous year was found to be an independent risk factor for new COPD diagnosis at visit 2 (HR, 3.92 [95% CI, 1.40 to 10.9], *p* = 0.002).

### 3.2. Incident Cardiometabolic Comorbidities

**Figure 2:**
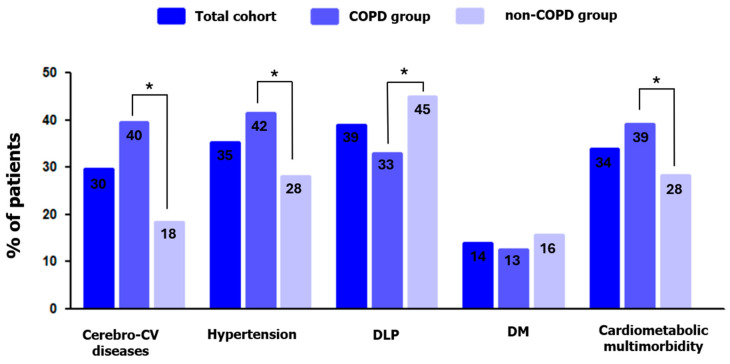
Percentage of patients who developed cardiometabolic comorbidities and cardiometabolic multimorbidity (≥2 cardiometabolic morbidities)) during the follow-up period. The figure shows percentages for the total sample (dark blue), for the sample grouped according to baseline diagnosis of COPD (moderate blue) or non-COPD (light blue). Cerebro-CV, cerebro-cardiovascular disease; DLP, dyslipidemia; DM, diabetes mellitus type II. * COPD vs. non-COPD = *p* < 0.05.

Among those participants who attended visit 2, the mean number of incident comorbidities for the total sample was 1.18 ± 1.01, and a third of them (n = 133; 34%) developed CMM. Figure 2 shows the frequency of incident cardiometabolic comorbidities (cerebro-cardiovascular diseases, hypertension, dyslipidemia, and diabetes mellitus) and CMM in the overall cohort and among subjects with COPD or non-COPD at baseline. Compared to subjects without COPD, patients with COPD at baseline were more likely to develop cerebro-cardiovascular diseases (40% vs. 18%, *p* < 0.001), systemic hypertension (42% vs. 28%, *p* = 0.006), and CMM (39% vs. 28%, *p* = 0.017).

There were 132 participants who developed CMM at visit 2. Compared with subjects who did not develop CMM, those in the subgroup who developed CMM were more likely to have been diagnosed with COPD and to be male (Table 2). They also had higher BMI and pack-year cigarette consumption and lower FEV_1_. There were more CMM developers who presented ≥ 1 hospital admission for respiratory problems in the year prior to the recruitment visit compared to non-CMM developers (58% vs. 46%; *p* = 0.02).

**Figure 3:**
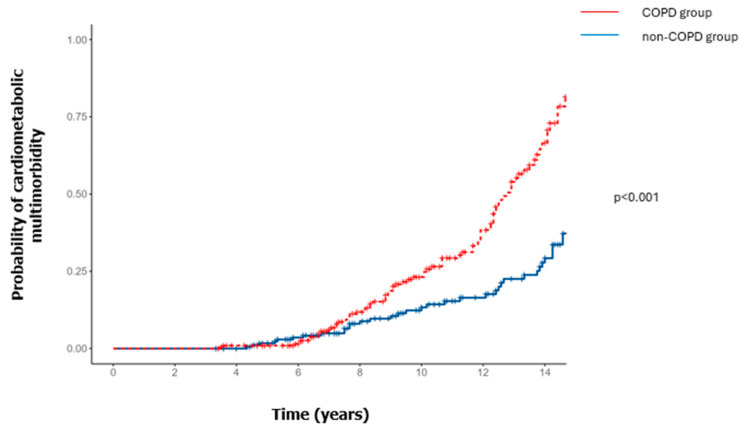
Cumulative incidence of cardiometabolic multimorbidity (≥2 incidents of cardiometabolic comorbidities, such as cerebro-cardiovascular events, hypertension, dyslipidemia, or diabetes). The assessment was a time-to-first-event analysis.

The cumulative incidence of CMM was 39% among patients with COPD vs. 28% among those without COPD (Table 2). Figure 3 presents the Kaplan–Meier survival curves for both COPD and non-COPD groups. The curves demonstrate that the probability of developing a CMM is significantly higher in the COPD group compared to the non-COPD group (log-rank test, *p* < 0.001). During the follow-up period, significant differences were observed in the incidence of the first episode of stroke (22% vs. 9%; *p* = 0.002) and peripheral arterial disease (11% vs. 0%; *p* = 0.02) between the COPD and non-COPD groups in the group of participants who developed at least one cerebro-cardiovascular event (Figure S1, Supplementary Materials).

Multivariable Cox analyses demonstrated that a positive COPD diagnosis at baseline (HR: 1.59, 95% CI: 1.03 to 2.46) and greater decliners (ΔFEV_1_ ≥ 40 mL/year) over the follow-up (HR: 1.57, 95% CI: 1.10 to 2.24) were independently associated with the development of CMM (Table 3). Specifically, COPD diagnosis at baseline (HR: 1.68, 95% CI: 1.08 to 2.62) and a history of at least one hospital admission due to respiratory symptoms in the year before the recruitment (HR: 2.42, 95% CI: 1.63 to 3.60) were associated with an increased occurrence of cerebro-cardiovascular events during the follow-up period. For incident systemic hypertension, COPD diagnosis was the only independent predictor (HR: 1.68, 95% CI: 1.12 to 2.52). While a COPD diagnosis at baseline was associated with a lower likelihood of developing dyslipidemia (HR: 0.66, 95% CI: 1.46 to 0.97), a more rapid decline in lung function over time was associated with an increased occurrence of this cardiometabolic comorbidity (HR: 1.55, 95% CI: 1.12 to 2.14). Regarding diabetes mellitus, a history of previous hospital admission was the sole predictor identified (HR: 1.97, 95% CI: 1.09 to 3.55) (Table 3).

## 4. Discussion

This long-term longitudinal study provides a comprehensive overview of the incidence of cardiometabolic conditions in smokers without any concomitant chronic morbid condition other than COPD. Our data indicated for the first time that, after nearly a decade of follow-up, the development of CMM was more frequent in smokers with a spirometry-based diagnosis of COPD at baseline and accelerated deterioration of lung function (≥40 mL/year in FEV_1_) than among smokers with no airway obstruction or slow pulmonary function decline.

Airway and systemic inflammation are closely linked to both the development of cardiometabolic comorbidities and accelerated lung function decline in COPD [23]. In smokers, persistent low-grade systemic inflammation resulting from ongoing exposure to cigarette irritants is associated with a cardiometabolic imbalance and increases the likelihood of cardiovascular events due to impaired endothelial function [24,25]. Additionally, chronic airway inflammation, also driven by the inhalation of cigarette irritants, is related to accelerated lung function decline [23]. Therefore, it is reasonable that both COPD and a more rapid decline in lung function are significant determinants of CMM in smokers without prior evidence of comorbidities. Future research should aim to better understand the underlying mechanisms of airway and systemic inflammation that lead to both accelerated lung function decline and the concurrent development of CMM in this population. Our results confirm previous studies that found an association between poorer lung function and a higher risk of developing several components of CMM. It has been reported that smokers with COPD have a 2- to 5-fold higher risk of incident coronary heart disease compared to non-COPD smokers [26]. In our study, this risk was determined to be twice as high after adjustment for shared risk factors such as sex, age, and BMI. A previous cross-sectional study has demonstrated a significant association between hypertension and COPD among adults aged less than 60 years [27]; however, this is the first report to show that the presence of COPD in smokers without additional comorbid conditions is also associated with a higher incidence of developing high blood pressure. Incident diabetes was not associated with baseline lung function in our cohort but with previous respiratory hospitalizations, a finding also reported in the COPDGene cohort [28]. These findings suggest that smokers newly diagnosed with COPD should be closely monitored for the potential development of cardiometabolic comorbidities, even if they initially do not present associated conditions. Future studies should prioritize the evaluation of preventive interventions for cardiometabolic comorbidities in newly diagnosed COPD patients, with particular emphasis on their effectiveness in reducing long-term adverse health outcomes.

The occurrence of incident CMM was observed in over one-third of the non-COPD smokers (28%) and in 39% of smokers with COPD. Furthermore, by performing lung function tests during the follow-up visit, we were able to demonstrate that smokers who experienced a greater decline in FEV_1_ over time showed a higher risk of developing CMM. While we only conducted two lung function assessments, which limits our understanding of lung function trends, this original finding is relevant and indicates the need to monitor the lung function of all smokers over time, especially those with COPD and without previous comorbidities. Data from the UK Biobank, a large cohort followed prospectively, showed that better lung function was associated with a lower risk for the development of CMM [29]. However, this study did not specifically evaluate the role of the presence or absence of a new COPD diagnosis, nor was there a longitudinal assessment of the decline in lung function [29]. Consequently, future studies should include more frequent lung function tests in smokers to validate our findings and better understand the relationship between lung function decline and CMM development.

Our study cohort, recruited entirely from a Smokers Clinic, may have a higher percentage of individuals with at least one respiratory-related hospital admission in the year prior to recruitment. Despite this selection bias, it is important to emphasize a key finding: having such a hospital admission for a respiratory condition is an independent risk factor for developing two cardiometabolic comorbidities, hypertension, and diabetes. These results align with previous studies that have emphasized the negative impact of having a history of respiratory events prior to a COPD diagnosis, which is associated with worse clinical outcomes [30,31]. This underscores the importance of establishing close monitoring protocols in clinical practice for these patients to identify and prevent the development of these comorbidities.

Worldwide, CMM has significant implications. CMM substantially increases the risk of death, reduces life expectancy in the general population [32], negatively impacts health outcomes, and increases the economic burden in people with COPD [4,14]. Given these serious consequences, the early identification of smokers at risk of developing CMM is a priority. This approach would allow for effective monitoring of future disease burden and emphasize the need for holistic management in this population.

Our study presents several key strengths. We followed a cohort of smokers for nearly a decade, all of whom started without any cardiometabolic chronic conditions. This long-term approach gave us a unique opportunity to explore lung function changes using spirometry tests and to prospectively record the incidence of CMM. Additionally, the study groups were well balanced in size, facilitating meaningful comparisons between them. Furthermore, all participants were treated in pulmonary clinics (COPD or smoker clinics) and therefore underwent treatments based on national and international consensus.

However, this study also has some limitations. First, although all participants with a new diagnosis of COPD were monitored in consultations and treated according to their clinical status, we do not have specific data on the therapy received during the follow-up period. Therefore, we did not fully adjust for medication use. However, there is no evidence that the available medications during the observed period meaningfully alter lung function decline or inhibit the development of cardiometabolic morbidities. Second, the lack of a standardized definition for CMM presents a challenge in comparing findings across different studies. For this reason, our analysis adopted an inclusive approach to CMM, incorporating not only the widely accepted core components—cerebro-cardiovascular events and diabetes—but also extending to include hypertension and dyslipidemia. However, it is relevant to note that some potential confounders (e.g., total cholesterol or LDL cholesterol) might also contribute to the incidence of cardiometabolic conditions in smokers. Since these lipid parameters are not typically included in current definitions of CMM, they were not explored in this study. Third, significant participant attrition occurred during the follow-up period, which may have impacted the results. The participants lost to follow-up were generally older and had greater lung function impairment at the initial visit. Consequently, the incidence of cardiometabolic conditions might have been higher in this group, potentially leading to an underestimation of the real frequency of the events. Finally, socioeconomic status and lifestyle-related habits (e.g., physical activity level or exercise) were not registered, despite their significant impact on the risk of developing chronic conditions.

In conclusion, our study shows that among smokers without concomitant comorbid conditions, the presence of COPD and an accelerated decline in lung function are associated with CMM. Future studies are required to explore whether the early identification of the development of CMM and its subsequent treatment delays the development of its major cardiometabolic consequences such as coronary heart disease and stroke.

## Figures and Tables

**Table 1:** Characteristics of participants stratified by a positive diagnosis of COPD at baseline (visit 1) *.

	All Participants (n = 391)	COPD (n = 207)	Non-COPD (n = 184)	*p* Value
Demographics				
Age (year)	56 (10)	61 (8)	51 (11)	<0.001
Male, No (%)	321 (82)	189 (91)	131 (71)	<0.001
BMI (kg/m^2^)	28.0 (4.6)	28.2 (4.2)	27.7 (5.1)	0.32
Active smokers, No (%)	173 (44)	75 (36)	98 (53)	0.002
Former smokers, No (%)	218 (56)	132 (64)	86 (47)	
Pack-years	41.0 (21.9)	47.4 (22.4)	33.9 (18.9)	<0.001
Lung function				
Post FEV_1_% predicted	80 (23)	66 (19)	96 (14)	<0.001
GOLD I (≥80% pred.), No (%)	-	46 (22)	-	-
GOLD II (50–79% pred.), No (%)	-	122 (59)	-	
GOLD III/IV (≤49% pred.), No (%)	-	39 (19)	-	
DL_CO_ % predicted	80 (21)	76 (22)	86 (18)	<0.001
Respiratory symptoms				
Chronic bronchitis, No (%)	152 (39)	82 (39)	70 (38)	0.55
Dyspnea (mMRC)	1.2 (0.8)	1.3 (0.9)	1.1 (0.8)	0.07
≥1 Hospital admission due to respiratory problems, No (%)	182 (35)	121 (40)	58 (27)	0.032
Pre-COPD **, No (%)			73 (40)	

* Data are expressed as No. (%) or mean (standard deviation, SD). ** Pre-COPD is defined as having normal spirometry results and either chronic sputum production, cough, or modified Medical Research Council (mMRC) score ≥ 2 points at baseline. COPD, chronic obstructive pulmonary disease; BMI, body mass index; FEV_1_, forced expiratory volume in the first second of forced spirometry; GOLD, the Global Initiative for Chronic Obstructive Pulmonary Disease; DL_CO_, diffusing capacity of the lungs for carbon monoxide.

**Table 2:** Baseline characteristics of participants who had incident cardiometabolic multimorbidity during the follow-up period *.

	Cardiometabolic Multimorbidity (n = 133)	No Cardiometabolic Multimorbidity (n = 258)	*p* Value
Spirometry status			
Non-COPD, No (%)	51 (28)	133 (51)	0.017
COPD, No (%)	81 (39)	126 (61)	
GOLD I (≥80% pred.), No (%)	19 (23)	27 (21)	
GOLD II (50–79% pred.), No (%)	48 (59)	74 (58)	
GOLD III/IV (≤49% pred.), No (%)	14 (17)	25 (20)	
Demographics			
Age (year)	59 (9)	55 (11)	<0.001
Male, No (%)	125 (94)	195 (75)	<0.001
BMI (kg/m^2^)	29.4 (4.3)	27.1 (4.5)	<0.001
Active smokers, No (%)	57 (43)	116 (45)	0.78
Former smokers, No (%)	76 (57)	142 (55)	
Pack-years	44.6 (23.1)	39.2 (21.0)	0.02
Lung function			
Post FEV_1_ % predicted	76 (21)	82 (24)	0.01
DL_CO_ % predicted	80 (20)	82 (20)	0.432
FEV_1_ rate of decline, mL/yr	37 (36)	33 (44)	0.411
Respiratory symptoms			
Chronic bronchitis, No (%)	53 (40)	99 (38)	0.71
Dyspnoea (mMRC)	1.4 (1.0)	1.2 (0.8)	0.13
≥1 Hospital admission due to respiratory problems, No (%)	77 (58)	119 (46)	0.02

* Data are expressed as No. (%) or mean (standard deviation, SD); COPD, chronic obstructive pulmonary disease; BMI, body mass index; FEV_1_, forced expiratory volume in the first second of forced spirometry; DL_CO_, diffusing capacity of the lungs for carbon monoxide.; mMRC, modified Medical Research Council.

**Table 3:** Multivariable Cox proportional hazard model of cardiometabolic multimorbidity and individuals’ cardiometabolic comorbidities (n = 391).

	Univariable		Multivariable (Adjusted) #	
	HR (95% CI)	*p* Value	HR (95% CI)	*p* Value
Cardiometabolic multimorbidity (≥2 cardiometabolic comorbidities)				
COPD diagnosis	1.76 (1.23 to 2.51)	0.002	**1.59 (1.03 to 2.46)**	**0.036**
Pack-years	1.01 (1.00 to 1.01)	0.049	1.00 (0.99 to 1.01)	0.613
Greater decliners (ΔFEV_1_ ≥ 40 mL/year)	1.30 (0.92 to 1.84)	0.131	**1.57 (1.10 to 2.24)**	**0.013**
≥1 Hospital admission due to respiratory problems	1.15 (0.82–1.63)	0.418	1.09 (0.76 to 1.57)	0.629
Cerebro-cardiovascular events				
COPD diagnosis	2.39 (1.60 to 3.57)	<0.001	**1.68 (1.08 to 2.62)**	**0.021**
Pack-years	1.01 (1.01 to 1.02)	<0.001	1.00 (1.00 to 1.01)	0.229
Greater decliners (ΔFEV_1_ ≥ 40 mL/year)	0.95 (0.65 to 1.38)	0.772	1.05 (0.72 to 1.55)	0.785
≥1 Hospital admission due to respiratory problems	2.27 (1.54 to 3.34)	<0.001	**2.42 (1.63 to 3.60)**	**<0.001**
Hypertension				
COPD diagnosis	2.40 (1.69 to 3.40)	<0.001	**1.68 (1.12 to 2.52)**	**0.012**
Pack-years	1.01 (1.00 to 1.01)	0.044	1.00 (0.99 to 1.00)	0.298
Greater decliners (ΔFEV_1_ ≥ 40 mL/year)	0.88 (0.62 to 1.24)	0.462	1.01 (0.71 to 1.44)	0.954
≥1 Hospital admission due to respiratory problems	0.82 (0.58 to 1.15)	0.248	0.80 (0.57 to 1.14)	0.217
Dyslipidemia				
COPD diagnosis	0.67 (0.48 to 0.92)	0.012	**0.66 (0.46 to 0.97)**	**0.035**
Pack-years	1.00 (0.99 to 1.01)	0.911	1.00 (1.00 to 1.01)	0.352
Greater decliners (ΔFEV_1_ ≥ 40 mL/year)	1.59 (1.16 to 2.19)	0.004	**1.55 (1.12 to 2.14)**	**0.008**
≥1 Hospital admission due to respiratory problems	0.97 (0.71 to 1.34)	0.871	0.90 (0.65 to 1.25)	0.537
Diabetes mellitus				
COPD diagnosis	0.81 (0.47 to 1.38)	0.432	0.82 (0.45 to 1.49)	0.505
Pack-years	1.00 (0.99 to 1.02)	0.507	1.00 (0.99 to 1.01)	0.868
Greater decliners (ΔFEV_1_ ≥ 40 mL/year)	1.33 (0.78 to 2.28)	0.291	1.41 (0.82 to 2.43)	0.211
≥1 Hospital admission due to respiratory problems	2.27 (1.28 to 4.02)	0.005	**1.97 (1.09 to 3.55)**	**0.024**

Clinically significant results are highlighted in bold. COPD, chronic obstructive pulmonary disease; FEV_1_, forced expiratory volume in the first second; HR, hazard ratio; CI, confidence interval. # Adjusted by age, sex, and body mass index.

## Data Availability

The data that support the findings of this study are available from the last author (J.M.M.) upon reasonable request.

## Supplementary Materials

The following supporting information can be downloaded at: https://www.mdpi.com/article/10.3390/jcm13247627/s1, Figure S1: Frequency of types of cerebro-cardiovascular events in the group of patients who developed at least one cerebro-cardiovascular event during the follow-up period (n = 122). COPD, chronic obstructive pulmonary disease; Cerebo-CV, cerebro-cardiovascular diseases. * COPD vs. non-COPD = *p* < 0.05; Table S1: Baseline characteristics of participants who attended or not attended visit 2; Table S2: Baseline characteristics of participants defined as greater decliner (ΔFEV1 ≥ 40 mL/year) or lesser decliner (ΔFEV1 < 40 mL/year) during follow-up *; Table S3: Baseline characteristics of Non-COPD participants at baseline who had COPD at visit 2 *.

## References

[1] Collaborators GBDCRD (2020). Prevalence and attributable health burden of chronic respiratory diseases, 1990–2017: A systematic analysis for the Global Burden of Disease Study 2017. Lancet Respir. Med..

[2] Chen S., Kuhn M., Prettner K., Yu F., Yang T., Barnighausen T., Bloom D.E., Wang C. (2023). The global economic burden of chronic obstructive pulmonary disease for 204 countries and territories in 2020–2050: A health-augmented macroeconomic modelling study. Lancet Glob. Health.

[3] Lokke A., Lange P., Lykkegaard J., Ibsen R., Andersson M., de Fine Licht S., Hilberg O. (2021). Economic Burden of COPD by Disease Severity—A Nationwide Cohort Study in Denmark. Int. J. Chron. Obstruct Pulmon Dis..

[4] Chen W., FitzGerald J.M., Sin D.D., Sadatsafavi M., Canadian Respiratory Research Network (2017). Excess economic burden of comorbidities in COPD: A 15-year population-based study. Eur. Respir. J..

[5] Tan D.J., Lodge C.J., Walters E.H., Bui D.S., Pham J., Lowe A.J., Bowatte G., Vicendese D., Erbas B., Johns D.P. (2024). Can We Use Lung Function Thresholds and Respiratory Symptoms to Identify Pre-COPD? A Prospective, Population-based Cohort Study. Am. J. Respir. Crit. Care Med..

[6] Agusti A., Celli B.R., Criner G.J., Halpin D., Anzueto A., Barnes P., Bourbeau J., Han M.K., Martinez F.J., Montes de Oca M. (2023). Global Initiative for Chronic Obstructive Lung Disease 2023 Report: GOLD Executive Summary. Eur. Respir. J..

[7] Dai X., Gil G.F., Reitsma M.B., Ahmad N.S., Anderson J.A., Bisignano C., Carr S., Feldman R., Hay S.I., He J. (2022). Health effects associated with smoking: A Burden of Proof study. Nat. Med..

[8] De Silva R., Silva D., Piumika L., Abeysekera I., Jayathilaka R., Rajamanthri L., Wickramaarachchi C. (2024). Impact of global smoking prevalence on mortality: A study across income groups. BMC Public Health.

[9] Divo M.J., Celli B.R., Poblador-Plou B., Calderon-Larranaga A., de-Torres J.P., Gimeno-Feliu L.A., Berto J., Zulueta J.J., Casanova C., Pinto-Plata V.M. (2018). Chronic Obstructive Pulmonary Disease (COPD) as a disease of early aging: Evidence from the EpiChron Cohort. PLoS ONE.

[10] Van Remoortel H., Hornikx M., Langer D., Burtin C., Everaerts S., Verhamme P., Boonen S., Gosselink R., Decramer M., Troosters T. (2014). Risk factors and comorbidities in the preclinical stages of chronic obstructive pulmonary disease. Am. J. Respir. Crit. Care Med..

[11] Fabbri L.M., Celli B.R., Agusti A., Criner G.J., Dransfield M.T., Divo M., Krishnan J.K., Lahousse L., Montes de Oca M., Salvi S.S. (2023). COPD and multimorbidity: Recognising and addressing a syndemic occurrence. Lancet Respir. Med..

[12] Sin D.D., Anthonisen N.R., Soriano J.B., Agusti A.G. (2006). Mortality in COPD: Role of comorbidities. Eur. Respir. J..

[13] Divo M., Cote C., de Torres J.P., Casanova C., Marin J.M., Pinto-Plata V., Zulueta J., Cabrera C., Zagaceta J., Hunninghake G. (2012). Comorbidities and risk of mortality in patients with chronic obstructive pulmonary disease. Am. J. Respir. Crit. Care Med..

[14] Huber M.B., Wacker M.E., Vogelmeier C.F., Leidl R. (2015). Excess costs of comorbidities in chronic obstructive pulmonary disease: A systematic review. PLoS ONE.

[15] Joseph J.J., Rajwani A., Roper D., Zhao S., Kline D., Odei J., Brock G., Echouffo-Tcheugui J.B., Kalyani R.R., Bertoni A.G. (2022). Associations of Cardiometabolic Multimorbidity With All-Cause and Coronary Heart Disease Mortality Among Black Adults in the Jackson Heart Study. JAMA Netw. Open.

[16] Celli B.R., Cote C.G., Marin J.M., Casanova C., Montes de Oca M., Mendez R.A., Pinto Plata V., Cabral H.J. (2004). The body-mass index, airflow obstruction, dyspnea, and exercise capacity index in chronic obstructive pulmonary disease. N. Engl. J. Med..

[17] Hajiro T., Nishimura K., Tsukino M., Ikeda A., Koyama H., Izumi T. (1998). Analysis of clinical methods used to evaluate dyspnea in patients with chronic obstructive pulmonary disease. Am. J. Respir. Crit. Care Med..

[18] Kim V., Criner G.J. (2013). Chronic bronchitis and chronic obstructive pulmonary disease. Am. J. Respir. Crit. Care Med..

[19] Macintyre N., Crapo R.O., Viegi G., Johnson D.C., van der Grinten C.P., Brusasco V., Burgos F., Casaburi R., Coates A., Enright P. (2005). Standardisation of the single-breath determination of carbon monoxide uptake in the lung. Eur. Respir. J..

[20] Miller M.R., Hankinson J., Brusasco V., Burgos F., Casaburi R., Coates A., Crapo R., Enright P., van der Grinten C.P., Gustafsson P. (2005). Standardisation of spirometry. Eur. Respir. J..

[21] Quanjer P.H. (1983). Standardized lung function testing: Report of the Working Party for the European Community for Steel and Coal. Bull. Eur. Physiopathol. Respir..

[22] Vestbo J., Edwards L.D., Scanlon P.D., Yates J.C., Agusti A., Bakke P., Calverley P.M., Celli B., Coxson H.O., Crim C. (2011). Changes in forced expiratory volume in 1 second over time in COPD. N. Engl. J. Med..

[23] Donaldson G.C., Seemungal T.A., Patel I.S., Bhowmik A., Wilkinson T.M., Hurst J.R., MacCallum P.K., Wedzicha J.A. (2016). Airway and Systemic Inflammation and Decline in Lung Function in Patients With COPD. Chest.

[24] Yanbaeva D.G., Dentener M.A., Creutzberg E.C., Wesseling G., Wouters E.F. (2007). Systemic effects of smoking. Chest.

[25] Delgado G.E., Krämer B.K., Siekmeier R., Yazdani B., März W., Leipe J., Kleber M.E. (2020). Influence of smoking and smoking cessation on biomarkers of endothelial function and their association with mortality. Atherosclerosis.

[26] Chen W., Thomas J., Sadatsafavi M., FitzGerald J.M. (2015). Risk of cardiovascular comorbidity in patients with chronic obstructive pulmonary disease: A systematic review and meta-analysis. Lancet Respir. Med..

[27] Liang X., Chou O.H.I., Cheung B.M. (2023). The Association Between Systemic Arterial Hypertension and Chronic Obstructive Pulmonary Disease. Results from the U.S. National Health and Nutrition Examination Survey 1999–2018: A Cross-sectional Study. Chronic Obstr. Pulm. Dis..

[28] Kinney G.L., Baker E.H., Klein O.L., Black-Shinn J.L., Wan E.S., Make B., Regan E., Bowler R.P., Lutz S.M., Young K.A. (2016). Pulmonary Predictors of Incident Diabetes in Smokers. Chronic Obstr. Pulm. Dis..

[29] Li G., Lu Y., Qiao Y., Hu D., Ke C. (2022). Role of Pulmonary Function in Predicting New-Onset Cardiometabolic Diseases and Cardiometabolic Multimorbidity. Chest.

[30] Whittaker H., Nordon C., Rubino A., Morris T., Xu Y., De Nigris E., Müllerová H., Quint J.K. (2023). Frequency and severity of respiratory infections prior to COPD diagnosis and risk of subsequent postdiagnosis COPD exacerbations and mortality: EXACOS-UK health care data study. Thorax.

[31] Ding B., Zaha R., Makita N., Graham S., Lambrelli D., Huse S., Müllerová H., Nordon C., Muro S. (2023). History of Respiratory Events Prior to a First COPD Diagnosis and Future Exacerbations: A Longitudinal Observational Cohort Database Study in Japan. Int. J. Chronic Obstr. Pulm. Dis..

[32] Fan J., Sun Z., Yu C., Guo Y., Pei P., Yang L., Chen Y., Du H., Sun D., Pang Y. (2022). Multimorbidity patterns and association with mortality in 0.5 million Chinese adults. Chin. Med J..

