# Supplementary material for: Incident Cardiometabolic Comorbidities in Smokers with/Without Chronic Obstructive Pulmonary Disease: A Long-Term Cohort Study
Source: J Clin Med. 2024 Dec 14;13(24):7627. doi: 10.3390/jcm13247627 (PMC11728173; doi:10.3390/jcm13247627)
Supplement: Supplementary file 1 [file jcm-13-07627-s001.zip › jcm-3349389-supplementary.pdf]

Title: Incident cardiometabolic comorbidities in smokers with/without Chronic Obstructive Pulmonary Disease: a long-term cohort study.

Supplementary material

Table S1. Baseline characteristics of participants who attended or not attended visit 2

	**Attended visit 2** (n = 391)	**Did not attend the visit 2** (n = 536)	P values
**Demographics**			
Age *(yr)*	56 (10)	63 (9)	< 0.001
Gender, male (%)	321 (82)	452 (86)	0.120
BMI *(kg/m^2)*	28.0 (4.6)	28.5 (4.9)	0.298
Active smokers, No (%)	172 (44)	179 (34)	< 0.001
Pack years	41.0 (21.9)	53.0 (25)	< 0.001
**Lung function**			
Post FEV_1 % predicted	80 (23)	62 (21)	< 0.001
DL_{CO} % predicted	81 (21)	67 (22)	<0.001
**Respiratory symptoms**			
Chronic bronchitis, No (%)	152 (39)	221 (42)	0.342
Dyspnoea *(mMRC)*	1.2 (0.8)	1.7 (1.1)	< 0.001

*Data are expressed as No. (%) or mean (standard deviation, SD).
COPD, chronic obstructive pulmonary disease; BMI, body mass index; FEV_1, forced expiratory volume in the first second of forced spirometry; DLCo, diffusing capacity of the lungs for carbon monoxide; mMRC, modified Medical Research council.

**Table S2. Baseline characteristics of participants defined as greater decliner ($\Delta FEV_1 \geq 40$ mL/year) or lesser decliner ($\Delta FEV_1 < 40$ mL/year) during follow-up***			
	**Greater decliner** (n = 159)	**Lesser decliner** (n=232)	P values
**Spirometry status**			
COPD, No (%)	79 (38.2)	128 (61.8)	0.286
Non-COPD, No (%)	80 (43.5)	104 (56.5)	
**Demographics**			
Age *(yr)*	56 (11)	56 (10)	0.784
Gender, male (%)	135 (42)	186 (58)	0.230
BMI *(kg/m²)*	28.2 (4.6)	28.4 (4.9)	0.683
Active smokers, No (%)	78 (49)	95 (41)	0.121
Pack years	41 (22)	40 (21)	0.703
**Lung function**			
Post FEV_1 % predicted	84 (20)	76 (23)	< 0.001
Post FEV_1 / FVC ratio	66 (14)	63 (16)	0.034
DL_{CO} % predicted	84 (20)	79 (20)	0.011
**Respiratory symptoms**			
Chronic bronchitis, No (%)	68 (43)	84 (36)	0.204
Dyspnoea *(mMRC)*	1.16 (0.08)	1.27 (0.89)	0.207
≥1 Hospital admission due to respiratory problems, No (%)	74 (46)	106 (45)	0.339
Pre-COPD at baseline, No (%)	31 (19.5)	42 (18.1)	0.728

*Data are expressed as No. (%) or mean (standard deviation).
Abreviations: COPD, chronic obstructive pulmonary disease; BMI, body mass index; FEV1, forced expiratory volume in the first second of forced spirometry; GOLD, the Global Initiative for Chronic Obstructive Pulmonary Disease, FVC, forced vital capacity; DLCO, diffusing capacity of the lungs for carbon monoxide; mMRC, modified Medical Research Concil; SD, standard deviation.

**Table S3. Baseline characteristics of Non-COPD participants at baseline who had COPD at visit 2***			
	**New COPD** (n = 29)	**Non-COPD** (n=155)	P values
**Demographics**			
Age *(yr)*	59 (9)	55 (11)	<0.001
Gender, male (%)	23 (79)	108 (70)	0.293
BMI *(kg/m^2)*	27.5 (5.5)	27.7 (5.0)	0.848
Active smokers, No (%)	17 (59)	81 (52)	0.551
Pack years	38 (29)	33 (30)	0.153
**Lung function**			
Post FEV_1 % predicted	92 (13)	98 (13)	0.034
DL_{CO} % predicted	82 (18)	87 (18)	0.178
FEV1 annual decline in mL/yr	61 (37)	33 (39)	<0.001
**Respiratory symptoms**			
Chronic bronchitis, No (%)	11 (88)	59 (38)	0.989
Dyspnoea *(mMRC)*	1.16 (0.18)	1.17 (0.22)	0.447
≥1 Hospital admission due to respiratory problems, No (%)	24 (83)	80 (52)	0.002

*Data are expressed as No. (%) or mean (standard deviation, SD).
COPD, chronic obstructive pulmonary disease; BMI, body mass index; FEV_1, forced expiratory volume in the first second of forced spirometry; DLCO, diffusing capacity of the lungs for carbon monoxide; mMRC, modified Medical Research Concil.

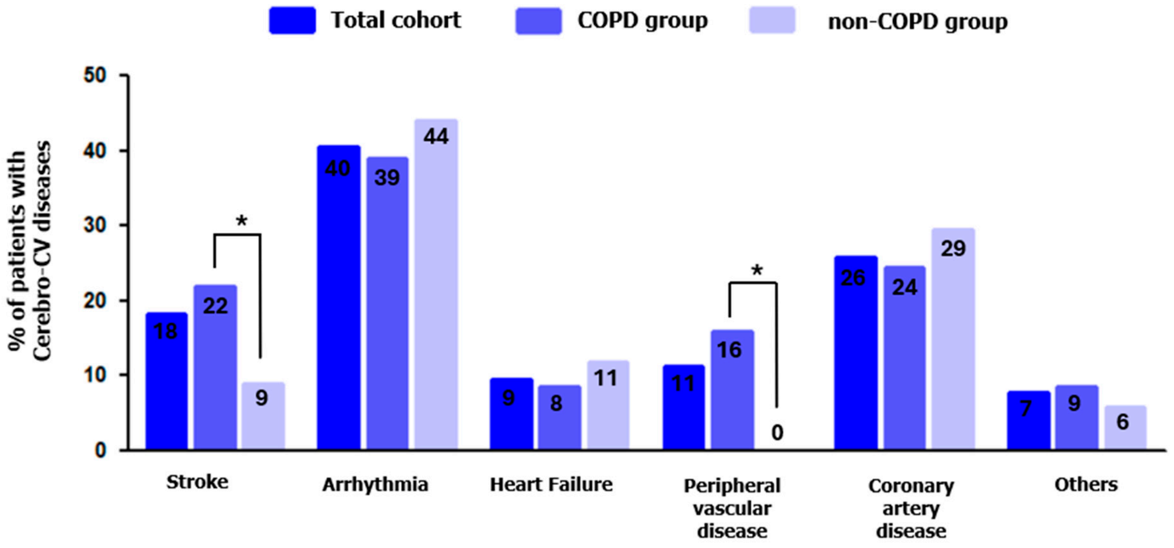

Figure S1. Frequency of types of cerebro-cardiovascular events in the group of patients who developed at least one cerebro-cardiovascular event during the follow-up period (n=122). COPD, chronic obstructive pulmonary disease; Cerebo-CV, cerebro-cardiovascular diseases. * COPD vs. non-COPD= $p< 0.05$
